# AZD8931, an equipotent, reversible inhibitor of signaling by epidermal growth factor receptor (EGFR), HER2, and HER3: preclinical activity in HER2 non-amplified inflammatory breast cancer models

**DOI:** 10.1186/1756-9966-33-47

**Published:** 2014-05-30

**Authors:** Zhaomei Mu, Teresa Klinowska, Xiaoshen Dong, Emily Foster, Chris Womack, Sandra V Fernandez, Massimo Cristofanilli

**Affiliations:** 1 Department of Medical Oncology, Thomas Jefferson University; and Kimmel Cancer Center, Philadelphia, PA 19107, USA; 2 Department of Medical Oncology, Fox Chase Cancer Center, Philadelphia, PA 19111, USA; 3 AstraZeneca, Macclesfield, Cheshire, UK; 4 Department of Surgical Oncology, First Affiliated Hospital, China Medical University, Shenyang, People’s Republic of China

**Keywords:** Inflammatory breast cancer, EGFR, HER2, HER3, AZD8931, Paclitaxel

## Abstract

**Introduction:**

Epidermal growth factor receptor (EGFR) overexpression has been associated with prognostic and predictive value in inflammatory breast cancer (IBC). Epidermal growth factor receptor 2 (HER2) overexpression is observed at a higher rate in IBC compared with noninflammatory breast cancer. Current clinically available anti-HER2 therapies are effective only in patients with HER2 amplified breast cancer, including IBC. AZD8931 is a novel small-molecule equipotent inhibitor of EGFR, HER2, and HER3 signaling. In this study, we investigated the antitumor activity of AZD8931 alone or in combination with paclitaxel using preclinical models of EGFR-overexpressed and HER2 non-amplified IBC cells.

**Methods:**

Two IBC cell lines SUM149 and FC-IBC-02 derived from pleural effusion of an IBC patient were used in this study. Cell growth and apoptotic cell death were examined *in vitro*. For the *in vivo* tumor growth studies, IBC cells were orthotopically transplanted into the mammary fat pads of immunodeficient mice. AZD8931 was given by daily oral gavage at doses of 25 mg/kg, 5 days/week for 4 weeks. Paclitaxel was subcutaneously injected twice weekly.

**Results:**

AZD8931 significantly suppressed cell growth of IBC cells and induced apoptosis of human IBC cells *in vitro*. Significantly, we showed that AZD8931 monotherapy inhibited xenograft growth and the combination of paclitaxel + AZD8931 was demonstrably more effective than paclitaxel or AZD8931 alone treatment at delaying tumor growth *in vivo* in orthotopic IBC models.

**Conclusion:**

AZD8931 single agent and in combination with paclitaxel demonstrated signal inhibition and antitumor activity in EGFR-overexpressed and HER2 non-amplified IBC models. These results suggest that AZD8931 may provide a novel therapeutic strategy for the treatment of IBC patients with HER2 non-amplified tumors.

## Introduction

Inflammatory breast cancer (IBC) is a rare and highly metastatic variant of breast cancer with the poorest survival of all types of breast cancer [1,2]. IBC has shown the capacity to spread early, primarily through lymphatic channels and secondarily through blood vessels causing the typical inflammatory clinical signs. Characteristic clinical symptoms are rapid onset and progression of breast enlargement with overlying skin changes, such as diffuse erythema, edema or peau d’orange, tenderness, hardening, and warmth; a tumor mass may or may not be present [3,4]. IBC primarily affects younger women under the age of 50 at diagnosis, and is difficult to be detected as most patients do not present with a lump, but rather occurs as tumor emboli. At the time of diagnosis, most patients have lymph node metastases, and 30% of the patients have distal metastases including brain, bones, visceral organs and soft tissue with variable frequency, in contrast to 5% of patients with non-IBC [5]. The lower survival rate of IBC patients may be due to the highly metastatic nature of the disease [6].

Primary treatment of patients with IBC is typically multimodal involving neoadjuvant combination chemotherapy followed by surgery, adjuvant chemotherapy, or radiotherapy [5]. The HER family has an important role in driving breast cancer. Epidermal growth factor receptor (EGFR) overexpression has been demonstrated as prognostic factors in IBC. Overexpression of epidermal growth factor receptor 2 (HER2) occurs during the stage of advanced tumor but whether the overexpression has a prognostic role in IBC has yet to be established [7,8]. Anti-HER2 therapies have shown benefit in IBC patients with HER2 amplification, which accounts for approximately 40% of IBC [9]. However, therapeutic options for patients with ER-negative and HER2 non-amplified IBC are very limited. IBCs are predominantly basal-like or triple-negative (TN) as characterized by the estrogen receptor (ER)-negative, progesterone receptor (PgR)-negative and HER2 non-amplified status [10]. EGFR is overexpressed in 30% of IBCs and 50% of TNIBCs [2,11]. IBC patients with EGFR-positive tumors have a lower overall survival rate than patients with EGFR-negative tumors, and EGFR overexpression in IBC is frequently associated with an increased risk of recurrence [9]. EGFR overexpression is also correlated with large tumor size, aggressiveness and poor prognosis [12,13]. Thus, EGFR could be a potential therapeutic target in IBC and, in particular, in patients with EGFR-overexpressed IBC that currently has very limited treatment options.

Currently there are few human IBC cell lines available for studying this complex disease. Although available cell lines were derived from IBC patients, the molecular signatures among IBC cell lines are very distinct. SUM149 was developed from the primary tumor of IBC patient, but *in vivo* xenograft model are unable to recapitulate the tumor emboli that are the signature of IBC in humans. We have recently developed a new IBC cell line, FC-IBC-02 that was derived from the pleural effusion fluid of a woman with secondary metastatic IBC [14,15]. FC-IBC-02 cells form tumor spheroids in suspension culture, a characteristic of cancer stem cells, and recapitulate the tumor emboli *in vivo* xenograft models. SUM149 and FC-IBC-02 could be different representative models for studying the biology of IBC, both SUM149 and FC-IBC-02 cell lines are basal-like and ER/Pgr(-), EGFR-overexpressed and HER2 non-amplified.

AZD8931 was developed with the hypothesis that combined inhibition of EGFR, HER2, and HER3-mediated signaling may be more effective for clinical cancer treatment [16]. Pharmacological profiling has shown that AZD8931 is a novel, equipotent, reversible small-molecule ATP competitive inhibitor of EGFR, HER2, and HER3 signaling. Previous results showed that AZD8931 was significantly more potent against EGFR, HER2 and HER3 signaling than other EGFR inhibitors such as lapatinib or gefitinib *in vitro*. AZD8931 has shown greater antitumor activity in a range of xenografted models compared with lapatinib or gefitinib [16]. In the present study, we examined the effects of AZD8931 on cell growth and apoptotic cell death of human IBC cells *in vitro*. Further, we investigated the antitumor activity of AZD8931 alone or in combination with paclitaxel in EGFR-overexpressed and HER2 non-amplified IBC models.

## Methods and materials

### Reagents and cell culture

AZD8931 was synthesized and generously provided by AstraZeneca [16]. SUM149 were obtained from Dr. Stephen Ethier (Kramanos Institute, MI, USA) and are commercially available (Asterand, Detroit, MI). SUM149 cells were cultured in Ham’s F-12 media supplemented with 10% fetal bovine serum (FBS), 1 μg/ml hydrocortisone, 5 μg/ml insulin and antibiotic-antimycotic. The FC-IBC-02 tumor cells were derived from primary human breast cancer cells isolated from pleural effusion of an IBC patient [14,15]. Human samples used in this study were acquired with approval of the Fox Chase Cancer Center’s Institutional Review Board. Importantly, written informed consent was obtained from each participant. FC-IBC-02 cells were cultured in DMEM/F12 media with 10% FBS and 1% L-glutamine and antibiotic-antimycotic.

### Antibodies and immunoblot

Following treatment with AZD8931 at the indicated concentration and time points, immunoblotting was performed as previously described [15]. In brief, cells were lysed in 1× lysis buffer (Cell signaling), and then the supernatant was collected by centrifuging at 10,000 rpm for 10 min at 4°C. Protein concentration was determined using the BCA protein assay reagent kit (Pierce, Rockford, IL). Equal amounts of protein from cell lysates were resolved by SDS-PAGE electrophoresis. The membranes were incubated at 4°C overnight with the following antibodies: mouse anti-EGFR (1:1000; Cell Signaling), rabbit anti-AKT and rabbit anti-phospho-AKT (1:1000; Cell Signaling), mouse anti-β-actin (1:5,000; Santa Cruz). After incubation with anti-mouse IgG horseradish peroxidase conjugated secondary antibody (1:5,000; Amersham Pharmacia Biotech), immunoreactive proteins were visualized by the enhanced chemiluminescence reagents.

### Cell proliferation and apoptotic assay

SUM149 and FC-IBC-02 cells (2 × 10^3^) were seeded in triplicate in a 96-well plate and cultured overnight. Cells were treated with AZD8931 at indicated concentration for 72 hrs. Cell proliferation was monitored at the indicated times, absorbance at 490 nm was measured using a microplate reader using the MTS assay (CellTiter 96 AQueous One Solution cell proliferation assay, Promega) according to the manufacturer’s instruction.

Apoptotic cells were measured by Annexin V staining. Cells (1 × 10^5^) were treated with 1 μM AZD8931 for 48 and 72 hrs. Cells were harvested and labeled with Annexin V-PE and 7-amino-actinomycin D (7-AAD) (Guava Technologies Inc, Burlingame, CA) according to the manufacturer’s instructions. The samples were then analyzed by Guava system on a GuavaPC personal flow cytometer (Guava Technologies).

### *In vivo* xenograft studies

The protocol was approved by FCCC institutional animal care and use committee (IACUC). SUM149 and FC-IBC-02 (3 × 10^6^) cells were suspended in 200 μL of 1:1 ratio of phosphate-buffered saline/matrigel (BD Biosciences) and orthotopically injected into the mammary fat pads of six week old female C.B-17 severe combined immunodeficient (SCID) mice. Tumor volume was calculated from the formula TV = L*W*H*0.5236 where L, W, and H are the tumor dimensions in three perpendicular dimensions by caliper measurement. When tumor volumes were approximately 50 mm^3^ for SUM149 cells or 80 mm^3^ for FC-IBC-02 cells, the mice were randomly allocated into four groups (5 mice per group) and treatments were initiated. AZD8931 was suspended in a 1% (v/v) solution of polyoxyethylenesorbitan monooleate (Tween 80) in deionized water and given once daily by oral gavage at 25 mg/kg for 4 weeks. Paclitaxel solution was diluted in saline and given twice weekly by subcutaneously injection at 10 mg/kg. The control-group received 1% Tween 80 vehicle treatment. Mice were sacrificed at 33 days (SUM149) or 26 days (FC-IBC-02) post treatments. Tumors were surgically removed and weighed.

### VeraTag analysis and immunohistochemical staining

Formalin fixed paraffin embedded sections of tumors from control animals were subjected to VeraTag™ analysis. A pair of antibodies, one conjugated to biotin and the other to a fluorescent molecule (VeraTag) suitable for analysis by capillary electrophoresis, bind to distinct epitopes on HER2, HER3 or PI3K. The VeraTag molecules are attached to the antibodies via photo-cleavable linkers. Methylene blue, conjugated to streptavidin, binds to the biotin-labeled antibody and is photo-activated by red-light. The released singlet oxygen, as a result of methylene blue catalyzed photosensitization, cleaves VeraTag molecules in close proximity to the antibody-biotin-streptavidin complex.

Tumor-bearing mice were treated with AZD8931 at 50 mg/kg/day for 4 days. Tumors were removed and fixed at 4 hrs after fourth dose. Formalin-fixed paraffin-embedded tumors were cut onto glass slides and processed for immunohistochemical (IHC) staining as previously described [16]. In brief, antigen retrieval was performed on formalin-fixed, paraffin-embedded tumor sections and the following primary antibodies were used: total EGFR (DAKO PharmDx), total HER2 (DAKO Herceptest), total HER3 (CST clone D43D4), phospho-EGFR (Epitomics #1139-1), phospho-HER2 (CST #2243), phospho-HER3 (CST #4791), A polymer detection system (DAKO Envision + K4007) was used for secondary detection and sections were counterstained with Carazzi’s hematoxylin. Semiquantitative scoring was carried out by light microscopy by a pathologist (CW) for immunohistochemical brown staining on a four point scale (0+, none; 1+, weak; 2+, moderate; 3+, strong) and for percentage (%) distribution, to calculate an H-Score (sum of 1 x% 1+, 2 x% 2+, and 3 x% 3+). Cytoplasmic and membrane staining was recorded.

### Statistical analysis

Quantitative data were expressed as mean ± SD. Analysis of variance (ANOVA) with Student’s *t* test was used to determine the significant differences among experimental groups, and *P* < 0.05 was considered significant.

## Results

### IBC xenografted tumors express low HER2 and low to medium HER3 levels

**Figure 1:**
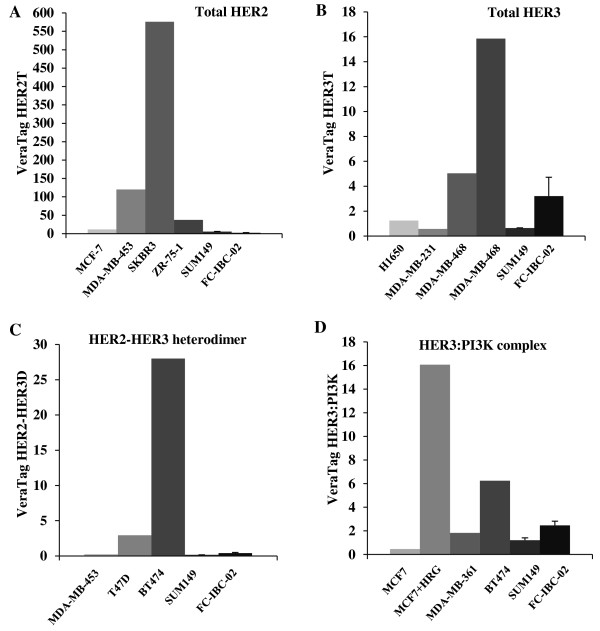
**IBC xenografted tumors express low HER2 and low to medium HER3 levels. A**. Total HER2, **B**. Total HER3, **C**. HER2-HER3 heterodimers, and **D**, HER3-PI3K complexes were measured in two xenografted tumor samples from each SUM149 or FC-IBC-02 cell lines by VeraTag™ technology. Normalized relative expression levels were compared with indicated breast cancer cell lines.

Both SUM149 and FC-IBC-02 overexpress EGFR and are HER2 non-amplified. However, the relative levels of HER2 and HER3 in these cell lines compared with other breast cancer cell lines were not known. We measured total HER2 and HER3 proteins, HER2-HER3 heterodimer and HER3-PI3K complex levels in xenografted tumor samples from SUM149 and FC-IBC-02 cells using the sensitive and quantitative VeraTag™ technology. When compared with samples from other breast cancer cell lines, total HER2 and HER2-HER3 heterodimers were expressed at low levels in both models (Figure 1A and C). Total HER3 and HER3-PI3K complexes were expressed at low levels in SUM149 xenografts and medium levels in FC-IBC-02 xenografts (Figure 1B and D). On the basis of these results, we conclude that IBC xenografted tumors express relatively low levels of total HER2 and HER2-HER3 heterodimers while the expression of HER3 and HER3-PI3K complexes is more variable across models, with the FC-IBC-02 model expressing moderate levels of these two complexes.

### AZD8931 inhibits EGFR pathway activity

**Figure 2:**
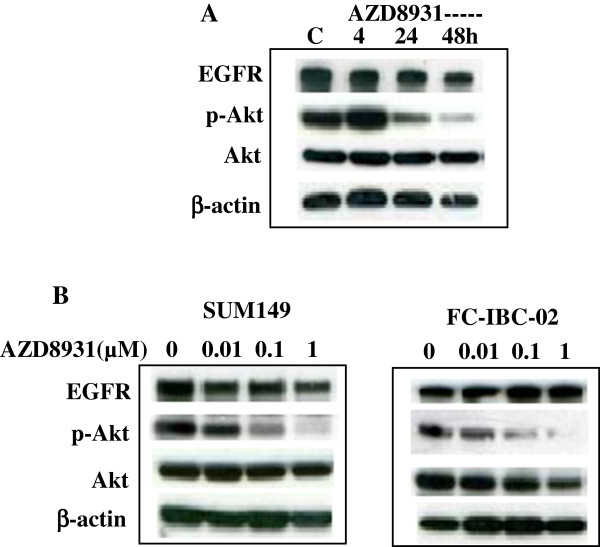
**AZD8931 inhibits EGFR pathway protein expression. A**. SUM149 cells were treated with vehicle control or 1 μmol/L AZD8931 for 4, 24, and 48 hrs. **B**. SUM149 and FC-IBC-02 cells were treated with 0 (vehicle), 0.01, 0.1, or 1 μmol/L AZD8931 for 24 hrs. Expression of EGFR, p-Akt, Akt, and β-Actin was examined by immunoblot analysis.

Previous study showed that AZD8931 is an equipotent, reversible inhibitor of EGFR, HER2 and HER3 signaling with potent *in vitro* inhibition of EGFR, HER2 and HER3 phosphorylation in breast cancer and squamous carcinoma cells [16]. As SUM149 and FC-IBC-02 cells express a high level of EGFR and low levels of HER2 and HER3, we sought to determine the effects of AZD8931 on the protein expression of EGFR and downstream markers. We tested the effects of AZD8931 on EGFR, phospho-Akt. in SUM149 cells at different time points. Western blot analysis showed that AZD8931 had no significant effect on EGFR expression level, and significantly inhibited phosphorylation of Akt in a time-dependent manner (Figure 2A). The inhibition of phospho-Akt was dose-dependent in both SUM149 and FC-IBC-02 cells (Figure 2B).

### AZD8931 inhibits proliferation and induces apoptosis in human IBC cells

**Figure 3:**
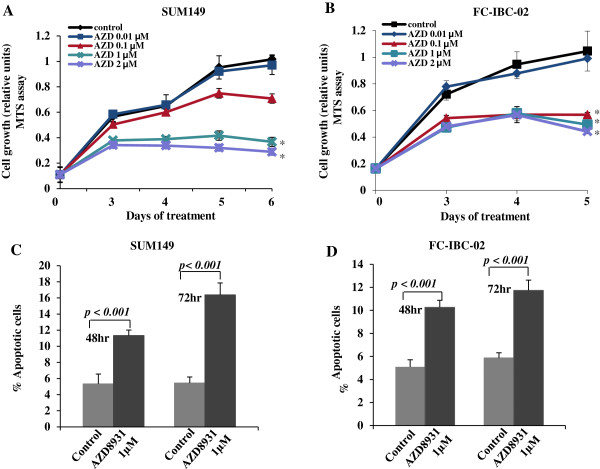
**AZD8931 inhibits proliferation and induces apoptosis in human IBC cells.** SUM149 **(A)** and FC-IBC-02 **(B)** cells were treated with 0.01, 0.1, 1, or 2 μmol/L AZD8931 for 72 hrs. At the indicated times, MTS assay was performed by absorbance at 490 nm. Mean of 3 independent experiments with SD. *P < 0.001 compared to control. **C** and **D**. SUM149 and FC-IBC-02 cells were treated with 1 μmol/L AZD8931 for 48 or 72 hrs. Annexin V-positive cells were measured by Guava Nexin assay. Mean of 3 independent experiments with SD. *P* value compared to control.

As previous study has shown that AZD8931 exposure results in significant inhibition of cell proliferation in squamous cell carcinoma of the head and neck and non–small cell lung carcinoma cell lines [16], we next sought to determine the effects of AZD8931 on IBC cell proliferation using the MTS assay. AZD8931 significantly suppressed the proliferation of SUM149 cells in a dose-dependent manner when compared with the control (Figure 3A). Similar suppression of proliferation by AZD8931 was observed for FC-IBC-02 cells (Figure 3B), suggesting that the observed effects were not cell line specific. Based on these results, we conclude that AZD8931 suppresses human IBC cell proliferation *in vitro*.

We next examined early apoptotic cell death by Annexin V staining. The percentage of apoptotic cell death was significantly higher when SUM149 and FC-IBC-02 cells were treated with AZD8931 at both 48 and 72 hrs (*P* < 0.001; Figure 3C and D), compared with controls.

### AZD8931 inhibits the tumor growth of human IBC models

**Figure 4:**
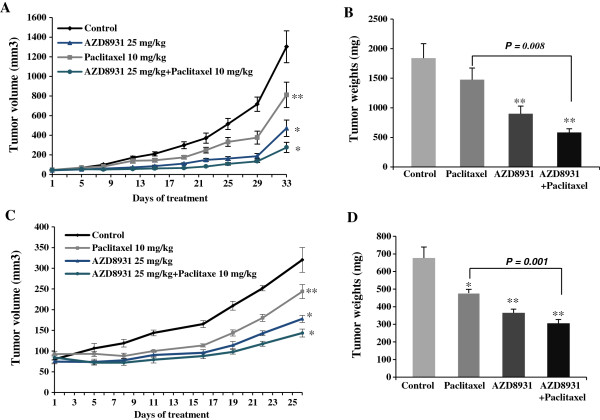
**AZD8931 inhibits the growth of SUM149 and FC-IBC-02 cells *in vivo *in SCID mice.** SUM149 **(A)** and FC-IBC-02 **(C)** cells were orthotopically transplanted into the mammary fat pads of SCID mice. Animals were randomized into groups (n = 5/group) when tumor volumes were approximately 50–80 mm^3^. AZD8931 was given by oral gavage at doses of 25 mg/kg per day, 5 days/week for 4 weeks. Paclitaxel was given twice weekly by subcutaneously injection at 10 mg/kg for 4 weeks. The mean tumor volumes were measured at the time points indicated. In SUM149 xenografts **(A)**, *P < 0.01 (vs. control), **P = 0.01 (vs. paclitaxel + AZD8931). In FC-IBC-02 xenografts **(C)**, *P < 0.001 (vs. control), **P < 0.01 (vs. paclitaxel + AZD8931). SUM149 **(B)** and FC-IBC-02 **(D)**, the size of tumors was measured by weights (mg) after tumors were removed from mice at the end of experiments. The data shown represent the mean of tumor weights with SD. *P < 0.05 (vs. control); **P < 0.01 compared to control. The combination of paclitaxel + AZD8931 compared with paclitaxel (*P* = 0.008, SUM149; *P* = 0.001, FC-IBC-02).

Previous study has shown that AZD8931 inhibits human tumor xenograft growth with different sensitivities to agents targeting either EGFR or HER2 in a variety of models including one human breast cancer cell line BT-474, which expresses ER/PgR, high levels of HER2, and moderate levels of EGFR [16]. Here, we determine the effects of AZD8931 alone or combined with paclitaxel on the growth of human IBC cells *in vivo* in SCID mice. Toward this goal, the tumors were orthotopically grown in the mammary fat pads of SCID mice and monitored by caliper measurement twice weekly. The changes in tumor volume following different treatments for both SUM149 and FC-IBC-02 cell lines are shown in Figure 4A and C. The tumor growth curves represent the group mean values over the course of 33 days for SUM149 xenograft and 26 days for FC-IBC-02 xenograft. AZD8931 alone significantly suppressed the xenografted tumor growth of SUM149 (*P* = 0.002; Figure 4A) and FC-IBC-02 (*P* < 0.001; Figure 4C) cells compared with the control group. The dose of AZD8931 at 25 mg/kg was chosen based on previous study [16]. Paclitaxel alone also delayed tumor growth over treatment compared with the control group in both xenografted human IBC models, but the effect of inhibition was much less than that seen in the AZD8931 alone group. The combination of paclitaxel + AZD8931 was more effective at delaying tumor growth than the control and other treatment groups in both xenografted IBC models. The difference was significant for paclitaxel + AZD8931 versus paclitaxel alone in SUM149 (*P* = 0.01; Figure 4A) and FC-IBC-02 (*P* < 0.01; Figure 4C). However, the difference was not statistically significant compared with AZD8931 alone.

**Figure 5:**
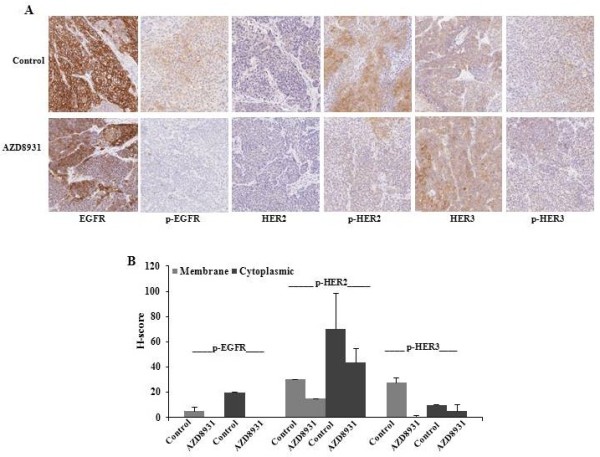
**AZD8931 inhibits EGFR pathway protein expression *in vivo*. A**. Immunohistochemical analysis of EGFR, HER2, HER3, phospho-EGFR, phospho-HER2, and phospho-HER3 in SUM149 xenografts following AZD8931 acute treatment (50 mg/kg/per day × 4 days). **B**. The average of pathologist’s H-score for both membrane and cytoplasmic staining.

In addition, we also examined the weight of xenografted tumors at the end of study. The inhibitory pattern of tumor size following different treatments was very similar to that seen in tumor growth curves in both IBC models. The combination of paclitaxel + AZD8931 was more effective at reducing tumor sizes than all of the other treatment groups. The difference was also significant for paclitaxel + AZD8931 versus paclitaxel alone in SUM149 (*P* = 0.008; Figure 4B) and FC-IBC-02 (*P* = 0.001; Figure 4D) models. Compared with AZD8931 alone, the difference was marginally significant for SUM149 tumors (*P* = 0.056) and FC-IBC-02 tumors (*P* = 0.07).Finally, we examined the expression of total EGFR, HER2, HER3, phosphorylated EGFR, phosphorylated HER2, and phosphorylated HER3 in SUM149 xenografted tumors by immunohistochemistry. As expected, high level expression of EGFR and low levels of HER2 and HER3 expression were observed in both AZD8931-treated and control tumors. The expression of phosphorylated EGFR, HER2, and HER3 was inhibited in AZD8931-treated tumors compared with control tumors (Figure 5A). The average of pathologist’s H-score for both membrane and cytoplasmic staining was shown in Figure 5B. Together, we conclude that AZD8931 significantly inhibits tumor growth in HER2 non-amplified IBC xenograft models by inhibiting EGFR, HER2 and HER3 phosphorylation. The combination of paclitaxel + AZD8931 was more effective than single agent paclitaxel or AZD8931 alone at delaying tumor growth.

## Discussion

In this study, we have shown that AZD8931 significantly suppressed IBC cell growth *in vitro* and tumor growth *in vivo* in two IBC cell lines including a new cell line-FC-IBC-02 derived from pleural effusion of an IBC patient. AZD8931 could have the potential to increase the antitumor activity when used in combination with chemotherapy.

EGFR can be overexpressed in all subtypes of breast cancer, but it is more frequently overexpressed in basal-like and triple-negative breast cancer including IBC [17-19]. A recent study showed that TNIBC is associated with poor overall survival and high locoregional relapse [20]. EGFR-positive IBC was associated with a significantly worse survival rate and increased risk of recurrence than EGFR-negative IBC [7,8]. There are several specific inhibitors of EGFR including gefitinib, erlotinib and cetuximab, and others have been studied for the treatment of breast cancer including IBC in clinical trials [21], but results so far remain controversial and disappointing. However, EGFR remains an important target for developing novel therapeutics because the options for TNIBC treatment are very limited.

Previous studies have shown that AZD8931 was significantly more potent in inhibiting cell growth *in vitro* and tumor growth *in vivo* across different cell line models including one human breast cancer cell line as compared with gefitinib or lapatinib [16]. AZD8931 also significantly affected EGFR, HER2, and HER3 phosphorylation and downstream signaling pathways, apoptosis, and proliferation. In the present study, we extended the previous study to further evaluate the antitumor activity of AZD8931 alone or in combination with paclitaxel in preclinical models of EGFR-overexpressed and HER2 non-amplified IBC. The SUM149 cell line expresses high levels of EGFR and is considered a representative IBC preclinical model, in spite of the fact it was developed from patients with primary disease, who had not yet received neoadjuvant therapy. The newly developed FC-IBC-02 cell line is a more representative model for the IBC studies, particularly for evaluating progression and metastasis, since the cell line has been developed from a patient with advanced IBC. FC-IBC-02 cells formed tumor spheroids and were able to develop tumor with the presence of tumor emboli and metastasis in SCID mice [14,15]. FC-IBC-02 cells expressed a high level of EGFR and relatively low levels of total HER2 and HER2-HER3 heterodimers making an ideal model to evaluate EGFR-targeting therapies. As expected, AZD8931 significantly inhibited cell proliferation *in vitro* and tumor growth of IBC cells *in vivo* in orthotropic xenografted models. Since FC-IBC-02 cells also expressed an intermediate level of HER3, AZD8931 could have potential to inhibit tumor growth through inactivation of HER2/HER3 and its downstream pathway. Previous study has shown that AZD8931 caused a significant increase of apoptotic protein expression in xenografted tumors [16]. Here, we showed a significant induction of apoptotic cell death following AZD8931 treatment *in vitro* in IBC cells.

Most significantly, in current IBC models, we showed that AZD8931 monotherapy significantly inhibited tumor growth and the combination of paclitaxel + AZD8931 resulted in the highest levels of tumor growth inhibition *in vivo* in both cell lines (Figure 4). The most common treatment for IBC is multimodal involving neoadjuvant combination chemotherapy followed by surgery, adjuvant chemotherapy, or radiotherapy [5]. Conventional chemotherapy regimens are not sufficient for the treatment of IBC, particularly for TNIBC. Targeted therapy against HER2 is one promising strategy for the treatment of IBC patients with HER2 amplification. Several EGFR targeted therapies including small molecule inhibitors and anti-EGFR antibodies have been evaluated in preclinical and clinical studies [21-25]. Patients with EGFR expressing tumors did not respond to EGFR-targeted therapy, which suggests that EGFR expression alone does not indicate tumor cell growth dependence on the EGFR pathway. One study indicated that the significant interactions between EGFR and other alternative signaling pathway kinases, such as c-MET and IGF-1R are linked to resistance to targeted therapies [26]. Thus, future studies are warranted to consider combining of EGFR-targeted therapy with drugs targeting other alternate signaling pathways to improve efficacy. Several antibodies targeting EGFR have also been investigated for their efficacy in patients with TNBC, some results have showed the clinical benefit in combination with chemotherapy drugs for patients with TNBC [27,28].

Metastasis is the primary cause of breast cancer mortality. IBC is characterized by locally advanced disease and high rates of metastasis even after multimodality treatments [29]. In IBC, inflammation is associated with the invasion of aggregates of tumor cells defined as tumor emboli, into the dermal lymphatics causing an obstruction of the lymph channels [30]. Currently, the molecular pathways driving the early development of metastasis in IBC remain poorly characterized. EGFR family and its downstream signaling pathways are known to promote cell migration, angiogenesis, invasion, and metastasis [22]. Previous studies have shown that the EGFR inhibitor erlotinib (Tarceva™) significantly inhibited cell motility, invasiveness, tumor growth, and spontaneous lung metastasis in EGFR-expressing IBC models [31]. Further therapeutic studies are warranted to examine the effects of AZD8931 on the invasiveness and metastasis of IBC.

## Conclusions

We demonstrate that EGFR/HER2/HER3-targeting with AZD8931 is associated with promising preclinical activity in EGFR-overexpressed and HER2 non-amplified IBC models, suggesting an important novel therapeutic approach for this aggressive disease.

## Abbreviations

IBC: Inflammatory breast cancer; EGFR: Epidermal growth factor receptor; HER2: Epidermal growth factor receptor 2; HER3: Epidermal growth factor receptor 3; TN: Triple negative.

## Competing interests

Teresa Klinowska, Emily Foster and Chris Womack are employees of and stockholders in AstraZeneca. All other authors declare that they have no competing interests.

## Authors’ contributions

ZM performed the experiments, analyzed the data and wrote the manuscript. TK, EF and CW assisted with immunohistochemical staining, analysed the data, reviewed and finalized the manuscript. XD and SF assisted with in vivo experiments. MC conceived of the study and finalized the manuscript. All authors read and approved the final manuscript.
